# Ca^2+^-Transport through Plasma Membrane as a Test of Auxin Sensitivity

**DOI:** 10.3390/plants3020209

**Published:** 2014-03-26

**Authors:** Anastasia A. Kirpichnikova, Elena L. Rudashevskaya, Vladislav V. Yemelyanov, Maria F. Shishova

**Affiliations:** 1 Department of Plant Physiology and Biochemistry, St. Petersburg State University, Universitetskaya emb. 7/9, St. Petersburg 199034, Russia; E-Mails: nastin1972@mail.ru (A.A.K.); bootika@mail.ru (V.V.Y.); 2 Department of Genetics and Biotechnology, St. Petersburg State University, Universitetskaya emb. 7/9, St. Petersburg 199034, Russia

**Keywords:** ABP1, auxin perception, Ca^2+^ signaling, elongation growth

## Abstract

Auxin is one of the crucial regulators of plant growth and development. The discovered auxin cytosolic receptor (TIR1) is not involved in the perception of the hormone signal at the plasma membrane. Instead, another receptor, related to the ABP1, auxin binding protein1, is supposed to be responsible for the perception at the plasma membrane. One of the fast and sensitive auxin-induced reactions is an increase of Ca^2+^ cytosolic concentration, which is suggested to be dependent on the activation of Ca^2+^ influx through the plasma membrane. This investigation was carried out with a plasmalemma enriched vesicle fraction, obtained from etiolated maize coleoptiles. The magnitude of Ca^2+^ efflux through the membrane vesicles was estimated according to the shift of potential dependent fluorescent dye diS-C_3_-(5). The obtained results showed that during coleoptiles ageing (3rd, 4th and 5th days of seedling etiolated growth) the magnitude of Ca^2+^ efflux from inside-out vesicles was decreased. Addition of ABP1 led to a recovery of Ca^2+^ efflux to the level of the youngest and most sensitive cells. Moreover, the efflux was more sensitive, responding from 10^−8^ to 10^−6^ M 1-NAA, in vesicles containing ABP1, whereas native vesicles showed the highest efflux at 10^−6^ M 1-NAA. We suggest that auxin increases plasma membrane permeability to Ca^2+^ and that ABP1 is involved in modulation of this reaction.

## 1. Introduction

One of the most frequent questions, which are faced by investigators of auxin effects is: How can this simple molecule trigger such a huge diversity of physiological reactions? During the last decades, an enormous amount of data on auxin signaling has accumulated. A big step in understanding the auxin mechanism of action was made after elucidation of the soluble hormone-receptor TIR1 and the closely related AFB proteins [1,2]. Nevertheless, not all auxin-induced reactions are mediated by activation of TIR1-signaling. A number of primary reactions, e.g., changes in membrane potential, cytosol acidification, elevation in cytosolic Ca^2+^ concentration, activation of PLA2, protoplast swelling, protein cycling, *etc*., require another type of receptor, localized in the plasma membrane [3,4,5,6,7]. Nevertheless, the process of auxin sensing at cell surface is still under investigation. The auxin binding protein 1 (ABP1) is considered to be a part of plasma membrane (PM) receptor or closely linked to it [7,8,9,10,11]. The ABP1 appears to be a dimer of 22-kDa subunit, and can easily be solubilized by detergent or acetone from membranes [12,13,14]. A number of *ABP1* genes are known to encode the protein in different plants [15,16,17,18,19]. The ABP1 protein has a single N-glycosylation site, which binds a mannose type glycan [14,20,21]. Two conservative domains (Box A, responsible for auxin binding, and Box B) and an ER targeting marker *C*-terminal KDEL tetrapeptide were determined in the ABP1 structure [21,22,23]. The main ABP1 pool is localized in ER where it is supposed to be inactive. Only about 2% of the protein is secreted to extracellular space to fulfill its physiological function [24].

It was supposed that ABP1 might be coupled to a transmembrane docking protein [25,26]. Unfortunately, up to now little is known about the nature and function of this protein. Based on the knowledge of different animal hormone-receptor complexes at the PM, several models were developed for the auxin receptor. The first one suggested that a kinase could fulfill the role of a transmembrane domain [27]. Another model implicates G-protein-coupled receptor as the auxin one [28]. The third model assumes that ABP1 binds/interacts with a Ca^2+^-permeable ion channel, which fulfills the role of a docking protein [29]. It is possible that ABP1 has more than one binding partner, differing in plant tissues and stage of development.

The absence of ABP1 or its reduction leads to significant changes: arrest of embryo development and elongation intensity [30,31]. Even heterozygous *ABP1*/*abp1* insertion mutants show a number of developmental disturbances confirmed by reduction of sensitivity to auxin and shift in the intensity of early auxin-regulated *Aux/IAAs* genes expression [32,33]. Decrease in ABP1 via antisense transformation leads to significant decrease in elongation intensity [31] and cell enlargement/protoplast swelling [34,35,36].

It was shown earlier that addition of exogenous ABP1 to a model system like protoplasts increased the amplitude of auxin-induced PM hyperpolarization [37]. Recently, a fast ABP1-related auxin-induced shift in the membrane potential (MP) was shown in a similar model system, by use of a sensitive fluorescent dye [38]. The advantage of the latter investigation was the ascertainment that the effect was triggered even by the *C*-terminal peptide of ABP1 and was blocked by antibodies against it. Overexpression of *ABP1* enhances the K^+^-transport by activation of K^+^-channels and quantity of their expression [39,40]. Thus, it could be concluded that ABP1 is an important modulator of cell sensitivity to the hormone at plasma membrane, but the mechanism of this regulation is still debated.

One of the fast and sensitive reactions triggered by auxin is an elevation of Ca^2+^ concentration in the cytosol. This reaction was estimated for different plant cells, including maize coleoptile parenchyma cells [9,41] Most probably it reflects the activation of plasma membrane channels, permeable for Ca^2+^ [9]. The coleoptile is a juvenile organ, the main function of which is to protect the first leaf at the initial stages of grass seedling development. Coleoptiles are very sensitive to auxin [42]. In maize coleoptiles, the native growth slows down tremendously from the 3rd to 5th day of seedling development [43]. The most intensive growth decrement appears at transition from the 3rd to 4th day of seedling development [44]. This phenomenon coincides with a loss of auxin-induced growth of coleoptile segments [43] and a significant decrease of auxin induced [Ca^2+^]_cyt_ elevation [44]. Thus, a possible reduction in cell sensitivity to the hormone is due to probable changes in auxin signal perception and early transduction. The current investigation focuses on the involvement of a plasma membrane Ca^2+^-transport system in auxin signal perception under the control of ABP1.

## 2. Results and Discussion

The intensity of Ca^2+^ transport through vesicle membranes, obtained from maize coleoptiles of different ages was estimated as ΔMP, determined by a shift in fluorescence of diS-C_3_-(5) dye, commonly used to test transmembrane potential not only in purified vesicles, but also at whole cell level, like protoplast or bacterial cell [45,46].

**Figure 1:**
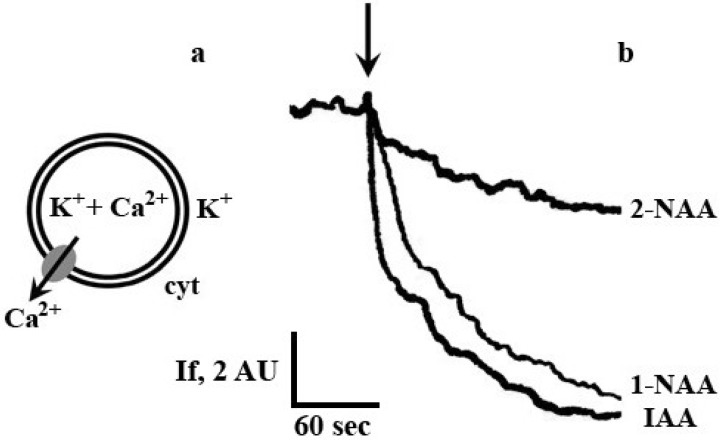
Auxin-induced generation of the membrane potential in a model system represented by plasma membrane vesicles from maize coleoptile cells isolated at the 3rd day of seedling development. (**a**) Scheme of vesicles loading (inside-out orientation of vesicle), cyt—cytosolic side of vesicle, arrow—direction of Ca^2+^ flux; (**b**) Single traces of diS-C_3_-(5) fluorescence shift after auxin addition, arrow—addition of auxin.

Our model system contained two types of vesicles: right-side-out, which copy the native cell orientation, and inside-out ones. Only Ca^2+^ ions had a gradient across the vesicle membrane (Figure 1a). Addition of IAA into the incubation medium led to a fast shift of dye fluorescence (Figure 1b), similar to our earlier results [47]. The detected shift in MP was due to Ca^2+^ efflux from the vesicles. We assume that right-side-out vesicles do not participate in ΔMP generation because transport of Ca^2+^ out of the cell is carried out by active systems like Ca^2+^-ATPase and by the Ca^2+^/proton antiporter systems (for review see [48]). Conditions for activation of these transporters were absent; therefore, the estimated ΔMP was due to flux of Ca^2+^ ions across membranes of inverted vesicles, which correspond to the flow directed into cell *in vivo*. The revealed IAA-induced shift in MP was similar to the effect obtained after addition of 1-NAA, an active synthetic auxin, but not after 2-NAA addition, a non-active synthetic analogue (Figure 1b). 

**Figure 2:**
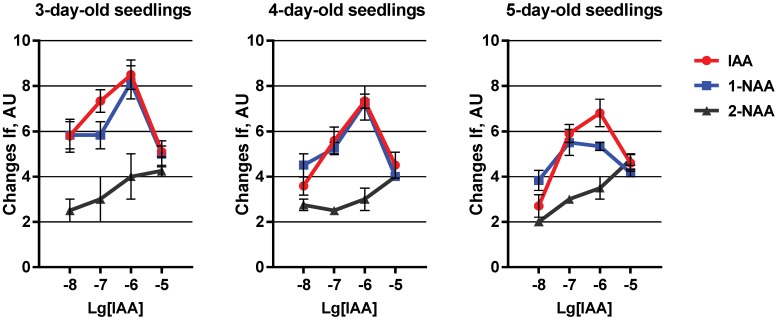
Auxin-induced generation of the membrane potential in a model system. Plasma membrane vesicles from maize coleoptile cells were isolated at the 3rd, 4th and 5th day of seedling development. Histograms represent mean values of the shift in diS-C_3_-(5) intensity of fluorescence (If) in arbitrary units (AU) ± SEM.

The fluorescence intensity of diS-C_3_-(5) is calibrated mainly in a model system with gradients of K^+^ and Na^+^ ions. The maximal amplitude of dye fluorescence in our model system decreases to −110 mV of the K^+^-diffusion potential as calculated by the Nernst equation when we added valinomycin into the Na^+^ incubation medium containing K^+^-loaded vesicles [47]. According to earlier results the IAA-induced change in dye fluorescence did not exceed −30 to 33 mV in case of membrane vesicles purified from coleoptiles at 4th day of seedling development. In the absence of Na^+^, detected changes of the amplitude of the auxin-induced fluorescence signal are presented in Figure 2 in arbitrary units.

The distinct auxin concentration dependence of the fluorescence difference was determined. In our experiments, IAA and 1-NAA caused a maximum effect by addition of hormone at 10^−6^ M (Figure 2). Similar results were obtained with vesicles isolated from seedling of all tested ages. However, the amplitude of the fluorescence signal decreased in vesicles from older seedlings. Only in the youngest seedlings the effect of IAA was slightly higher than that of 1-NAA. The 2-NAA-induced shift of diS-C_3_-(5) fluorescence increased slightly but almost linearly with concentration. The reaction did not exceed 4 arbitrary units (Figure 2). Moreover, no difference was obtained for 2-NAA during seedling ageing. Thus we conclude that this reaction is auxin-specific.

The obtained results show that physiologically active native and synthetic auxins trigger transport of Ca^2+^ through plasma membrane. The magnitude of this transport decreases within seedlings ageing. That coincides well with earlier results on protoplasts from maize-coleoptile cells, which showed a decrement of [Ca^2+^]_cyt_ elevation after auxin addition correlated with age of the coleoptile [44]. All this points out a possible decrease of auxin sensitivity at the plasma membrane and early transduction steps, like cytosolic Ca^2+^ elevation, over ageing [44]. If the idea is correct, that ABP1 is an important component of a hormone receptor at the plasma membrane then addition of this protein to the model system might restore the sensitivity for auxin lost within ageing.

**Figure 3:**
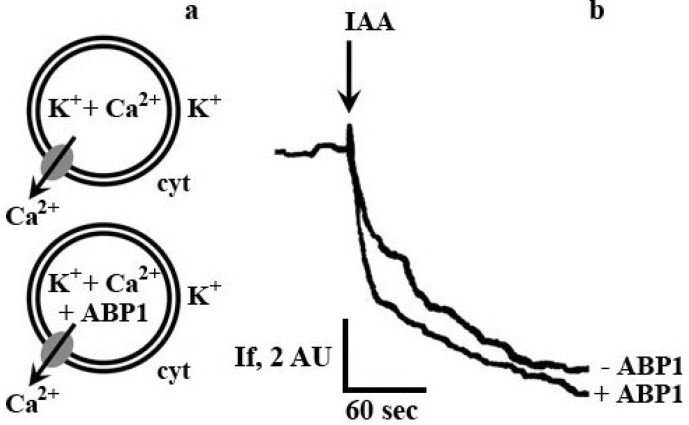
Effect of auxin binding protein 1 (ABP1) on auxin-induced generation of the membrane potential in a model system represented by plasma membrane vesicles from maize coleoptile cells at the 3rd day of seedling development. (**a**) Scheme of vesicles loading (inside-out orientation of vesicle), cyt—cytosolic side of vesicle, arrow—direction of Ca^2+^ flux; (**b**) Single traces of diS-C_3_-(5) fluorescence shift after auxin addition.

Therefore, we modified the model system by loading of ABP1 (10^−9^ M) into the vesicles (Figure 3a). Addition of IAA 10^−6^ M to this system did not change the dynamics of fluorescent response and almost did not affect the amplitude in case of the youngest seedlings (Figure 3b). 

**Figure 4:**
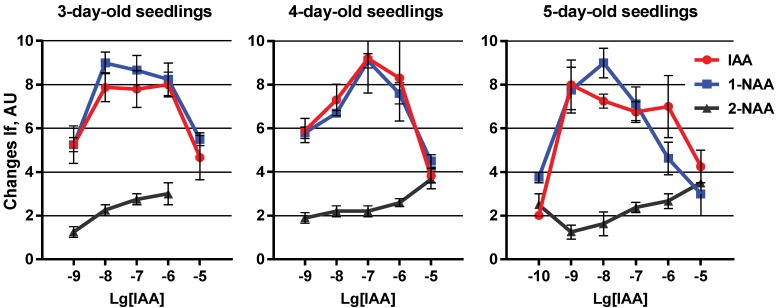
The role of ABP1 in determination of auxin-induced generation of the membrane potential in a model system, represented by plasma membrane vesicles from maize coleoptile cells at the 3rd, 4th and 5th day of seedling development. Histograms represent mean values of the shift in diS-C_3_-(5) intensity of fluorescence (If) in arbitrary units (AU) ± SEM.

The presence of ABP1 inside the vesicles led to significant changes in concentration dependence of the response. IAA and 1-NAA-triggered Ca^2+^ efflux at the 3rd day of development did not increase the maximum amplitude but it reached a maximum sensitivity at 10^−8^ M (Figure 4). Thus, we found a significant increase in PM sensitivity to auxins. 

A change in auxin sensitivity was shown in older seedlings at 4th and especially at 5th day of seedling development (Figure 4). However, in none of the cases did ABP1 addition increase the maximum value of ΔMP generated in vesicles obtained at the 3rd day of seedling development, when the sensitivity to the hormone had its highest value. Thus, it might be concluded that ABP1 is a limiting factor, which determines the sensitivity of the plant cell at the cell surface. A developmental decrease in ABP1 concentration may occur with seedling ageing toward the end of coleoptile physiological function, and may be coincided with a slowdown of the Ca^2+^ transport through the plasma membrane. Supplementary ABP1 restored the amplitude of Ca^2+^ transport and, as we assume, increased sensitivity to the hormone.

Special attention was paid to ABP1 because recently more evidences indicate an important role of this protein in the perception of the auxin signal at the plasma membrane. In a number of electrophysiological investigations done on tobacco leaf protoplasts maize ABP1 and antibodies against it strongly affected sensitivity to auxin [26,27,49]. Addition of ABP1 to protoplasts from plants transformed with *rol* genes of *Agrobacterium rhizogenes* raised sensitivity to auxin 100- to 1000-fold. It was supposed that ABP1 might increase the number of active perception units at plasma membrane, which shift protoplast sensitivity [27]. Both stimulatory effects of ABP1 and inhibitory effects of antibodies to this protein were also found in another electrophysiological model system—the whole cell patch clamp [50]. ABP1 mediated an auxin-induced shift in cytosolic pH and a flux of K^+^ [51,52]. Recent investigations showed that overexpression of ABP1 enhanced sensitivity of guard cells to auxin [40] and affected enlargement and division of plant cells [31,53,54].

Besides, the whole ABP1 protein, also its binding domain, as represented by the surface of an antibody D16 having auxin activity and the *C*-terminal peptide of ABP1 have physiological activity. D16 was able to trigger MP hyperpolarisation [55] and stimulation of the anion channel [37,56] in the absence of auxin. On the other hand, a synthetic peptide containing 12 residues of the *C*-terminus of ABP1, could mimic the action of high auxin concentrations in regulation of the K^+^ current, MP value and cytosol alkalinization in guard cells [38,51,52]. Thus, the suggestion was that this peptide played an important role in auxin-ABP1 coupling to intracellular signal cascades [57]. 

All listed results indicate the importance of ABP1 at the plasma membrane level but does not reveal the mechanism of its action. Recent publications frequently assume that ABP1 participates in regulation of endocytosis [58,59]. Endomembrane trafficking is a process of great importance, which maintains the intracellular re-localization of macromolecules and membranes by secretion and endocytosis. In plant cells traffic of newly synthesized proteins, translocation to the endoplasmic reticulum (ER), and subsequent protein processing and targeting occur via vesicle trafficking through the secretory pathway. Vesicle secretion is a very important process in plant cell growth and signaling [60]. Several publications showed that during auxin-dependent formation of lateral roots, the endomembrane system plays an important role, establishing cellular localization and polarity of the auxin transporters [61,62,63]. Polarity of auxin transporters leads to the formation of auxin gradients determining both initiation of lateral root and the establishment of the primordium cell patterning [64,65]. According to the recently accepted opinion, ABP1 effects are due to ROP GTPase signaling [59]. Nevertheless, the mechanism of transition of auxin signal from extracellular ABP1 to ROP-signaling in the cytosol is still under investigation.

In this manuscript we present data on a fast auxin activation of Ca^2+^ transport through the plasma membrane. This effect is paralleled by the increase of cytosolic Ca^2+^ concentration found before [44]. The latter event might trigger another process, namely vesicle secretion. A fast initiation of exocytosis by elevation of [Ca^2+^]_cyt_ is well known for neurons and now is shown for plant cells [66,67,68,69]. Even if taking into consideration that exocytosis in animal cells is much more sensitive to cytosolic Ca^2+^ in comparison to cereal coleoptile protoplasts, the phenomenon might indicate common mechanisms of signaling. Analysis of electronic micrographs of oat coleoptile cells reveals a ~12% difference in number of vesicles which are correlated with alterations in growth capacity of the tested cells, preferentially in cells which start elongation [70]. We assume the secretory mechanism is the same in cells from the same tissue, whereas the pool size of vesicles may vary from cell to cell in identical cell types. Elevation of [Ca^2+^] induced by auxin is estimated to be in the range of approximate 100 nM after auxin treatment [71,72]. This is enough to activate the secretory system within 10–20 s after the increase in Ca^2+^ concentration [66]. 

**Figure 5:**
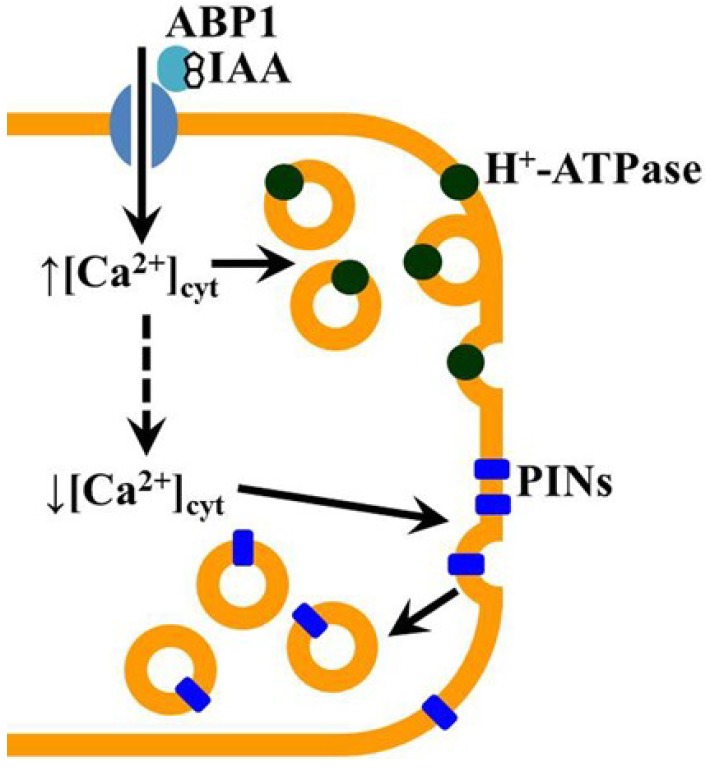
Hypothetical scheme of auxin perception at the plasma membrane and primary signal transduction events in coleoptile cells at early stages of development. In physiologically young coleoptile cell auxin interacts with extracellular ABP1 and triggers Ca^2+^ transport inside the cell ([72], data presented here). Cytosolic Ca^2+^ elevation might initiate exocytosis [66]. This will result in the increase in H^+^-ATPases protein in the plasma membrane [73]. Further Rho-like GTPase from plants (ROPs) signaling cascade, which is suggested to be linked to ABP1 [78], leads to endocytosis and redistribution of plasma membrane (PM) proteins, including auxin transporters PINs.

The importance of exocytosis in the mechanism of auxin action nicely correlates with an earlier suggestion about hormone-induced increase in a number of H^+^-ATPases in plasma membrane via vesicle secretion [73], which will lead along with post-transcriptional phosphorylation [74] to membrane hyperpolarization, shift in ion transport through plasmalemma, increase in acidification of cell wall and cell extension further on. The amplitude of Ca^2+^ rise might be considered as a threshold. Our data show that it depends on the amount of ABP1 and the external concentration of Ca^2+^ ([72], data presented here). Exocytosis is usually accompanied by recycling of vesicles. Electron micrograph data shows that about 60% of delivered vesicles to plasma membrane are recycled [75]. The intensity of recycling will depend on ROP GTPase signaling [76] and concentration of the cytosolic Ca^2+^ concentration [77]. Recently, ROP GTPase signaling was suggested to be linked to ABP1 [78] in support to our model (Figure 5). If cells already pass a step of elongation and have a loss or significant decrease in ABP1 perception facility then this could be another reason for the suggested scheme modulation. 

## 3. Experimental

### 3.1. Plant Material

Maize seedlings (*Zea mays* L., cv. Moldavsky-215) were grown in darkness at 26 ± 1 °C and 95% relative humidity. Seedlings were illuminated for 4 min every 24 h with a weak light in order to get straight coleoptiles and decrease mesocotyl growth. Seedlings were grown 48 h on wet filter paper and were thereafter transferred on glass tube raft on 1/10 Chesnokov nutrient solution. The ratios between different organs of the seedlings (leaf: coleoptile: mesocotyl: root) in cm were approximately 1.4:1.9:1.6:4.4; 2.3:4.2:2.9:9.1; 7.5:4.9:5.6:10.4, for 3-, 4- and 5- day-old seedlings, respectively. 

### 3.2. Measurement of Calcium Transport Rate across Plasma Membrane Vesicles from Maize Coleoptile Cells

The fraction enriched with plasma membranes was isolated from the decapitated coleoptiles of 3, 4 and 5-day-old etiolated maize seedlings. The membrane fraction was prepared by differential centrifugation with subsequent purification in a sucrose density gradient [47]. The purified membranes were collected at the interface between sucrose layers of 1.13 and 1.17 g/cm^3^ density. Inhibitor analysis showed no appreciable contamination with vacuolar membranes. The membrane fraction contained vesicles of right-side-out and inside-out orientation in the proportion 1:1, according to the enzymatic test with alamethicin. 

Membrane vesicles were loaded by osmotic shock with a medium containing 150 mM K_2_SO_4_, 1 mM Tris-MES, 150 mM sucrose, pH 6.8 (K^+^-medium) or with 1 mM CaCl_2_ in addition (Ca^2+^ + K^+^-medium). ABP1 10^−9^ M, was added to the loading medium depending on the experiment scheme. After loading, the vesicles were concentrated by centrifugation (99,500× *g* for 1 h). The pellet was re-suspended in the K^+^-medium. 

The change in membrane potential (MP), generated by ion transport through the membrane vesicles due to Ca^2+^ ion gradients, was recorded by the change in fluorescence intensity of 3,3'-dipropylthiodicarbocyanine iodide (diS-C_3_-(5)) fluorescent probe (Molecular Probes, Eugene, OR, USA) [79]. This dye is highly sensitive and can be used at a low concentration (0.8 μM). The fluorescence intensity is proportional to MP in a wide range. Fluorescence was measured with a spectrofluorometer constructed similar to MPF44-a Hitachi-Perkin-Elmer model (for details see [47]). Experiments were performed with a standard 1-cm quartz cuvette (volume of 0.3 mL). Excitation and emission wavelengths were 570 and 668 nm, respectively. Fluorescence was measured at an angle of 90 to the direction of excitation light. Aliquots of the dye solution and vesicle preparations were added to the cuvette with K^+^-incubation medium. Auxins were added after mixing the membrane fraction with the incubation medium and reaching a steady level in MP.

Experiments were performed in 6–7 replicates in 3–4 independent assays. Figures are made with GraphPad Prism 6 and represent mean values and standard error of the mean (SEM) for *n* = 4–8.

## 4. Conclusions

In summary, we would like to suggest the following hypothetical scheme for auxin perception at the plasma membrane and further primary transduction (Figure 5). The sensitivity of coleoptile cells to auxin is determined by the amount of ABP1, which is postulated to vary during elongation growth. At high external Ca^2+^ concentration auxin induces a fast (10–20 s) elevation of the cytosolic concentration of this ion. In young cells, containing a vesicle pool, this may lead to activation of exocytosis. A further intensification of H^+^-ATPase activity, a shift in membrane potential and magnitude of ion transport, cell wall acidification and initiation of elongation growth is supposed to occur. The cytosolic concentration of Ca^2+^ decreases rapidly and, in turn, auxin may induce a shift in endocytosis activity, which is followed by redistribution of PIN proteins and further formation of a new local auxin gradient. 

## Abbreviations

ABP1: auxin binding protein 1
AFB: auxin F-box protein
AU: arbitrary units
[Ca^2+^]_cyt_: concentration of Ca^2+^ in cytoplasm
cyt: cytosol
diS-C_3_-(5): 3,3'-dipropylthiodicarbocyanine iodide
ER: endoplasmic reticulum
IAA: indole-3-acetic acid
MP: membrane potential
1-NAA: naphthalene-1-acetic acid
PLA2: phospholipase A2
PM: plasma membrane
SEM: standard error of the mean
TIR1: transport inhibitor resistant 1
